# Supplementary material for: Development of a computer-aided design software for the quantitative evaluation of aesthetic damage
Source: PLoS One. 2019 Dec 18;14(12):e0226322. doi: 10.1371/journal.pone.0226322 (PMC6919621; doi:10.1371/journal.pone.0226322)
Supplement: S1 Code site — 1. (ZIP) [file pone.0226322.s001.zip › [EESC_jr][Projeto]Relatorio_07_21/reports/templates_zip/backyard-high-converting-landing-page-template.zip!/backyard/backyard/privacy.html]

Privacy Policy Backyard Landing Page


## Privacy Policy

Please read these Terms carefully, and contact us if you have any questions.

Epsum factorial non deposit quid pro quo hic escorol. Lorem ipsum dolor sit amet, consectetur adipiscing elit. Cras augue lorem, convallis in fringilla in, gravida eu leo. Donec porta commodo elit, id tincidunt nunc vestibulum vel. Cras ante leo, tincidunt eu lacus eget, malesuada dictum enim.

- Epsum factorial non deposit quid pro quo hic escorol. Lorem ipsum dolor sit amet, consectetur adipiscing elit. Epsum factorial non deposit quid pro quo hic escorol. Lorem ipsum dolor sit amet, consectetur adipiscing elit.
- Epsum factorial non deposit quid pro quo hic escorol. Lorem ipsum dolor sit amet, consectetur adipiscing elit. Epsum factorial non deposit quid pro quo hic escorol. Lorem ipsum dolor sit amet, consectetur adipiscing elit.
- Epsum factorial non deposit quid pro quo hic escorol. Lorem ipsum dolor sit amet, consectetur adipiscing elit. Epsum factorial non deposit quid pro quo hic escorol. Lorem ipsum dolor sit amet, consectetur adipiscing elit.
- Epsum factorial non deposit quid pro quo hic escorol. Lorem ipsum dolor sit amet, consectetur adipiscing elit. Epsum factorial non deposit quid pro quo hic escorol. Lorem ipsum dolor sit amet, consectetur adipiscing elit.
- Epsum factorial non deposit quid pro quo hic escorol. Lorem ipsum dolor sit amet, consectetur adipiscing elit. Epsum factorial non deposit quid pro quo hic escorol. Lorem ipsum dolor sit amet, consectetur adipiscing elit.

Epsum factorial non deposit quid pro quo hic escorol. Lorem ipsum dolor sit amet, consectetur adipiscing elit. Cras augue lorem, convallis in fringilla in, gravida eu leo. Donec porta commodo elit, id tincidunt nunc vestibulum vel. Cras ante leo, tincidunt eu lacus eget, malesuada dictum enim.

Back to Main Page


Copyright Backyard 2015
